# Gastric cancer with brain metastasis: from molecular characteristics and treatment

**DOI:** 10.3389/fonc.2024.1310325

**Published:** 2024-03-21

**Authors:** Yingze Zhu, Miao Zhou, Congling Li, Wenyue Kong, Yuning Hu

**Affiliations:** ^1^ Department of Oncology, Harbin Medical University Cancer Hospital, Harbin, China; ^2^ Department of Oncology, Tang Shan Central Hospital, Tangshan, China; ^3^ School of Clinical Medicine, Affiliated Hospital, North China University of Science and Technology, Tangshan, China

**Keywords:** gastric cancer, brain metastasis, molecular characteristics, immune checkpoint inhibitors (ICIs), immune-related adverse events (irAEs)

## Abstract

Gastric cancer is one of the cancers with increasing incidence and ranks fourth globally among the most frequent causes of cancer-related mortality. Early gastric cancer is often asymptomatic or presents with atypical symptoms, and the majority of patients present with advanced disease upon diagnosis. Brain metastases are present in approximately 1% of gastric cancer patients at the time of diagnosis, which significantly contributed to the overall mortality of the disease worldwide. Conventional therapies for patients with brain metastases remain limited and the median overall survival of patients is only 8 months in advanced cases. Recent studies have improved our understanding of the molecular mechanisms underlying gastric cancer brain metastases, and immunotherapy has become an important treatment option in combination with radiotherapy, chemotherapy, targeted therapy and surgery. This review aims to provide insight into the cellular processes involved in gastric cancer brain metastases, discuss diagnostic approaches, evaluate the integration of immune checkpoint inhibitors into treatment and prognosis, and explore the predictive value of biomarkers in immunotherapy.

## 1 Introduction

**Figure 1:**
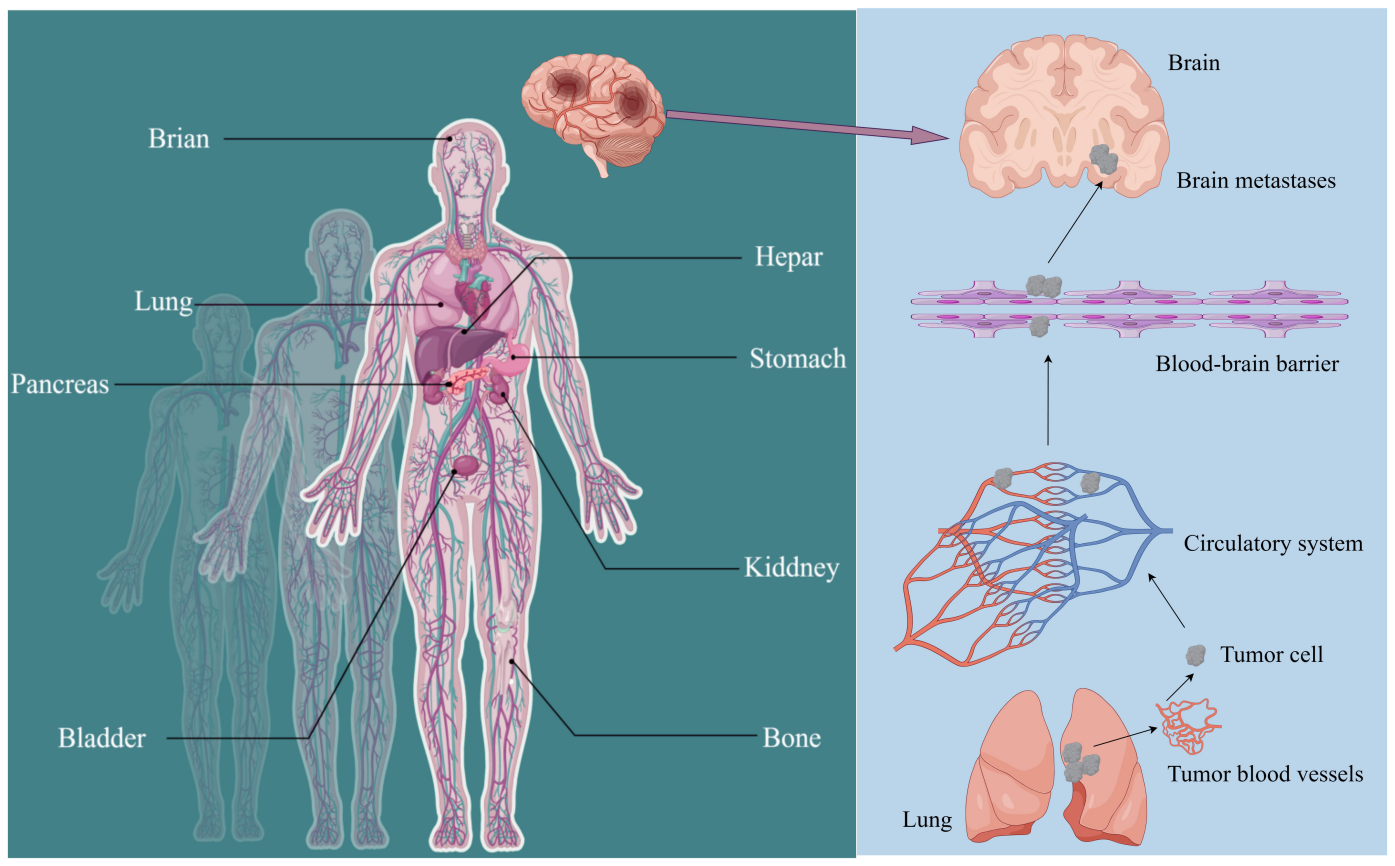
Metastatic sites of gastric cancer (GC) and detailed routes underlying tumor metastasis to the brain. The common metastasis sites of GC include the stomach, hepar, colorectum, kiddney, lung, brain, bladder, pancreas, and bone. Gastric cancer cells invade and penetrate tumor vasculature to enter the bloodstream. Upon surviving within the circulatory system, they subsequently breach the blood-brain barrier and other protective barriers to colonize and form metastatic foci in the brain.

Gastric cancer (GC) is among the top five cancers worldwide in terms of incidence and mortality (1). According to the latest global cancer data from the World Health Organization (WHO), the majority of newly diagnosed gastric cancer patients each year are mainly from Southeast Asian countries. In China alone, approximately 500,000 individuals are initially diagnosed with gastric cancer (2). Advanced gastric cancer has limited treatment options and efficacy, resulting in a median overall survival (mOS) of only 8 months (3). Furthermore, gastric cancer can metastasize to various sites in the body. The liver, lungs, bones, brain, and kidneys are frequent metastatic targets in advanced gastric cancer. Notably, the incidence of brain metastasis in patients with advanced gastric cancer represents a significant hazard, critically endangering both patient health and survival (**Figure 1**) (4).

For patients with gastric carcinoma in situ, subendothelial mucosal dissection and mucosal resection can effectively control and partially cure the disease, with 5-year survival rate exceeding 90% (5). Additionally, both open surgery and laparoscopic surgery can be beneficial for patients with GC. However, the probability of developing brain metastases increases with timely diagnosis, abundant treatment and longer patient survival (6). Therefore, it is crucial to elucidate the molecular mechanisms of brain metastases from gastric cancer in order to improve survival rates. While brain metastases account for only 1% of distant metastases and may present with common symptoms such as personality changes, dizziness, headache, cerebral oedema and brain herniation, they can be fatal to patients (7).

Gastric cancer is classified into two main subtypes based on the site of occurrence: cardia-associated gastric cancer and non-cardia gastric cancer (8). Additionally, depending on the age of diagnosis, gastric cancer is divided into early-onset gastric cancer and conventional gastric cancer, with a cut-off of 45 years. Researchers have classified gastric adenocarcinoma into various histological subtypes according to the 2019 World Health Organization (WHO) Classification of Tumors of the Digestive System (9). The most common histological subtype is tubular adenocarcinoma, which often forms fungal-like masses in the gastric lumen. Papillary adenocarcinoma is also frequently encountered in clinical practice and often metastasizes to adjacent organs such as the liver. Mural cell carcinomas are notable for having a more abundant eosinophilic granular cytoplasm. Mucinous adenocarcinoma is characterized by the the presence of large amounts of extracellular mucus, and accounts for approximately 10% of gastric cancers. In addition, hypoadhesive carcinomas are highly aggressive against lymphatic vessels and often show diffuse growth of cancer cells, such as indolent cell carcinoma. In addition to the above subtypes, there is also a mixed type of adenocarcinoma of the stomach, which includes several different histological components and has a poorer prognosis than patients with only one component (10). This type is closely associated with the development of brain metastases (11).

Gastric cancer patients with brain metastases have a lower median overall survival (mOS) of only approximately 5.3 months (2-9.6 months) in comparison to other cancers, such as lung, breast and kidney cancers (12). The prognosis of gastric cancer with brain metastases is also influenced by the presence of other metastatic sites, such as liver and lung metastases, and the progression of systemic disease (13). However, it is a complex and multi-stage process that involves the epithelial mesenchymal transition (EMT) of cancer cells, enabling them to spread to other organs via the bloodstream. Ghojazadeh et al. (14) have shown that KRAS mutation is an independent pathogenic factor for brain metastasis in GC. In addition, the level of SERPINH1 in gastric tissues of GC patients is higher than normal gastric mucosal tissues, and this factor is closely related to the levels of MMP-2, MMP-9, E-calcineurin and N-calcineurin. Of these factors, the association between SERPINH1 and EMT is closely related to brain metastasis (15–17).

With the ongoing evolution of treatments and protocols, traditional treatment options such as surgery, chemotherapy and targeted therapy have considerably improved the clinical outcomes for patients diagnosed with GC. Chemotherapy is one of the conventional treatments for advanced GC patients. Common chemotherapeutic agents include paclitaxel (doxorubicin or paclitaxel), capecitabine, fluorouracil (18). However, the clinical benefits are limited by the potential risk of injury from surgery, the toxicity of chemotherapeutic agents, and resistance to targeted drugs. The median survival time of patients with advanced GC treated with conventional therapy is less than 1 year (19). However, the field of tumor immunotherapy has made remarkable breakthroughs in both research and clinical practice, offering new hope to GC patients. Several immunotherapy regimens have achieved good efficacy in treating GC. In this article, we will review the latest clinically relevant advances in GC treatment, with a particular emphasis on the recent progress in immunotherapy.

## 2 Mechanism of GC brain metastasis

**Figure 2:**
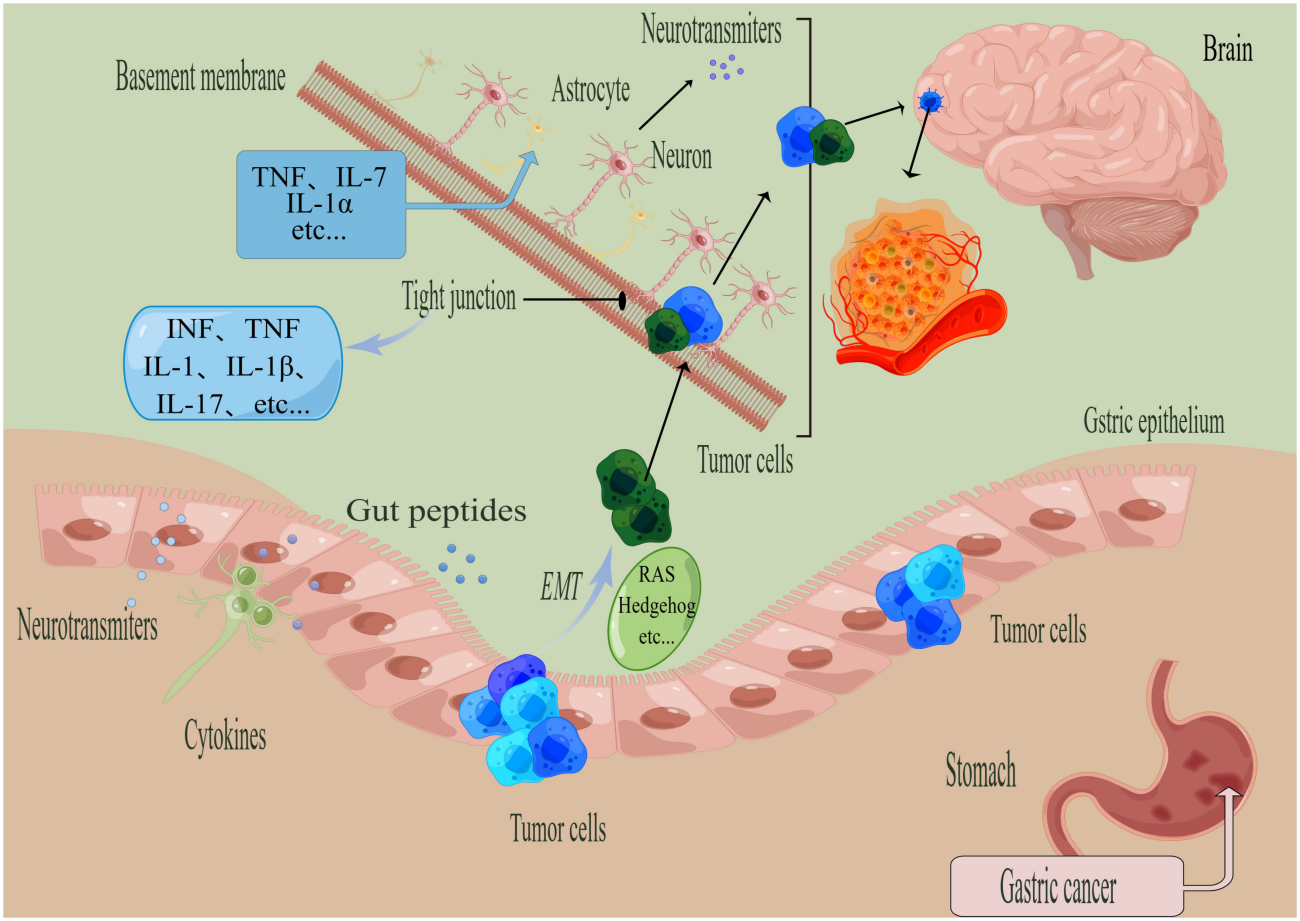
General steps of the metastasis of gastric cancer (GC). (By Figdraw). The process of tumor metastasis involves four steps, namely invasion, entry into the circulatory system, breaking through the BBB, and colonization. The EMT process is regulated by various factors, such as RAS, Hedgehog, and others. When tumor cells metastasize to the brain, the tumor cells need to cross the tight junction and other structures. Microglia activate astrocytes to upregulate TNF through the action of TNF-α, IL-1β, IL-1α, and other proinflammatory factors. Factors that can disrupt tight junctions include IL-1, IL-17, IL-1β, IFN, TNF, among others.

The complexity of mechanism in GC brain metastasis lies in the multi-stage process (**Figure 2**). The study of brain metastasis not only takes into account the interaction between immune cells such as cancer cells, pericytes, glial cells and macrophages, but also microenvironmental factors such as hypoxia (20).

### 2.1 EMT and local invasion

Upon achieving a certain size and undergoing angiogenesis, tumor foci *in situ* enter the critical stage of metastasis, during which tumor cells acquire advanced mutational capabilities for local invasion (21). GC cancer cells leave the primary cancer sites and attack the surrounding tissues, a hallmark of this stage being their breach of the basement membrane, ultimately leading to the progression of malignant tumors.

**Figure 3:**
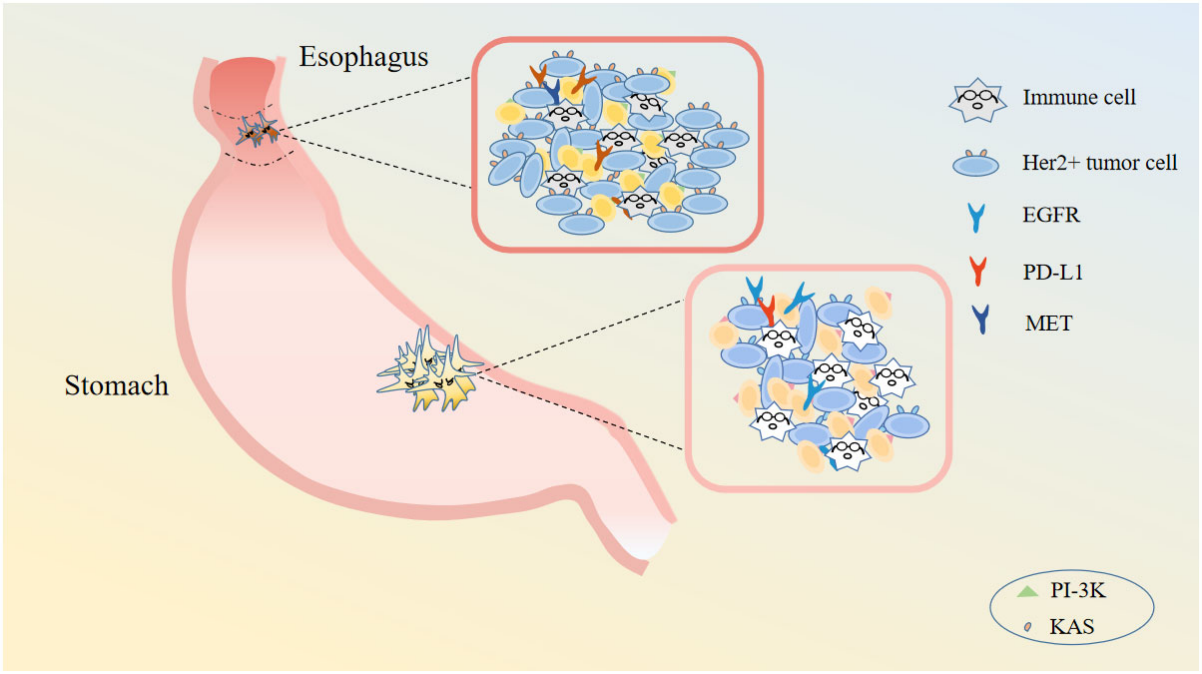
Gastric cancer cell related immune cells and targets. Gastric cancer cell concludes immune cells, Her2+ tumor cells and so on. There are many targets for gastric cancer: EGFR, PD-L1, MET, and so on.

The local invasion of GC tumor cells is closely related to the process of epithelial to mesenchymal transition (EMT) and neuron specific enolase (NSE). During EMT, epithelial-like cancer cells transform to migratory and invasive mesenchymal-like cancer cells, acquiring a variety of characteristics such as self-renewal, self replication, differentiation and invasion of stroma (22). Neuroendocrine differentiation characterizes GC, and NSE, an enzyme specific to neuroendocrine cells, is a predictive and sensitive biomarker in GC (23). Studies have shown that down-regulating the expression of NSE gene can increase the expression of NM23 and E-cadherin, thereby inhibiting the proliferation and invasion of GC. Additionally, activation of Wnt/β-catenin signaling pathway has been shown to promote the EMT process of GC cells also, thereby influencing their invasiveness (15). Numerous studies have revealed that the induction of EMT in GC involves several signaling pathways, such as transforming growth factor-β (TGF-β), Ras, Notch, Wnt, RTK, ETAR, hedgehog and matrix metalloproteinases (MMPs) (24–26) (**Figure 3**).

### 2.2 Molecular signalling cascades and circulation

Both GC patients and GC mouse models commonly experience distant metastasis through blood or lymphatic pathways, which are crucial for the development of brain metastases in GC.

The cascade of molecular signals generated between the primary tumor and brain metastases facilitates the specific spread of metastatic tumor cells through the lymphatic or hematogenous circulation. Bassey-Archibong et al. (27) analyzed the transcription profiles of different stages of the transfer cascade and found that brain metastasis initiating cells (BMICs) of cancer cells are captured before visible brain metastases are formed. These cells exhibit stem cell-like characteristics and can easily survive in the brain. RNA sequencing indicated that increased expression of HLA-G in BMICs prior to metastasis could promote the formation of brain metastases through the HLA-G/SPAG9/STAT3 axis. In addition, Cheng et al. (28) showed that SNHG1 downregulation inhibited metastasis of GC cells through the miR-195-5p/YAP1 axis, thereby suppressing the development of brain metastases. In addition, TGF-β also plays an important role in tumor metastasis and acquisition of immune escape. Han et al. (29) suggested that loss of the original characteristics of the TGF-β-smad signalling pathway due to Smad3 deficiency can promote tumor metastasis. Intracranial metastasis of GC can occur through the above molecular mechanisms with lymphatic origin, often metastasizing to the meninges and presenting as soft meningeal carcinoma (30). Tumor cells can metastasize through both the lymphatic and haematological pathways, but often at an advanced stage (31). In addition, BMICs must undergo transendothelial migration (TEM) to cross the brain endothelium and endothelial junctions (32). Sakurai et al. (33) suggested that primary lesion cancer cells invade the blood vessels and return to the right heart via the portal system, eventually colonizing brain tissue. The specific metastasis of gastric cancer cells to the brain depends on the bidirectional selection of the vasculature and is related to the anatomical system. García-Gómez et al. suggested that cancer cells and cerebrovascular endothelial cells can be mediated by integrins, L1CAM, FGF receptors, and ErbB receptors (34, 35).

However, the mechanisms for the survival of cancer cells in the circulatory system are relatively more clear for breast cancer and melanoma than for GC. The key factors that mediate the interaction between the GC cells and the plasma coagulation system in brain metastasis remain elusive. Unravelling the detailed molecular mechanisms involved in this process is essential for successful prevention of GC brain metastasis.

### 2.3 Destruction of barriers

The brain barrier acts as a crucial gatekeeper for the central nervous system, safeguarding the brain from inflammatory mediators. It is formed by a highly selective, semipermeable border of endothelial cells in the brain, which effectively prevents the entry of biological agents and other potential risk factors that may cause damage.

#### 2.3.1 BBB

The blood brain barrier (BBB) is a tight barrier structure that separates blood from brain tissue. It is composed of various cell types, including endothelial cells, astrocytes, podocytes, pericytes, microglia, oligodendrocytes and neurons (36, 37). The blood-brain barrier, with its stringent architecture and selective permeability, serves as a protective shield for both children and adults, safeguarding the brain’s microenvironment and the central nervous system’s stability (38).

The integrity of the BBB is often compromised by metastasized cancer cells, which are able to break through the tight junction structure, damage the capillary basement membrane, tight junctions, and increase pore size, distance between blood vessels, and permeability (39). To breach the BBB, cancer cells employ a complex network of molecular mechanisms that are closely linked to astrocytes, the most abundant cell type in the central nervous system (40, 41). Upon contact with circulating tumor cells in the brain, astrocytes transform into reactive astrocytes that influence cancer cell activity by secreting matrix metalloproteinases (MMPs) (42), which are important proteases that regulate the microenvironment of brain metastasis. Study has demonstrated that zinc can upregulate the expression of claudin-5 in neuronal cells, which in turn influences the permeability of the blood-brain barrier (43). Among the MMPs, MMP-2 and MMP-9 show the strongest correlation with patient survival, followed by MMP-1 (44). Studies indicate that astrocytes play a critical role in modulating the protein and matrix composition of tumor cells by releasing MMP-2 and MMP-9, thereby promoting the invasion and metastasis of tumor cells in the brain. In the MDA-MB-435 brain metastasis mouse model, researchers observed increased MMP-9 expression during the invasion of cancer cells (45). MMP inhibitors have been shown to decrease tumor invasion and metastasis. Meanwhile, astrocytes have been found to affect cell morphology and cell cycle by secreting heat-sensitive lipid epoxyeicosatrienoic acids (EETs) during the process (46).

#### 2.3.2 BCSFB

The blood cerebral spinal fluid barrier (BCSFB) is formed by the choroid plexus and acts as a barrier between plasma and cerebrospinal fluid. This barrier is also crucial in maintaining the stability of the brain microenvironment.

Recent studies have demonstrated that the BCSFB prevents the entry of tumor cells into the cerebrospinal fluid or arachnoid (47). The maintenance of the inner environment of the brain and spinal cord by the BCSFB is primarily dependent on the tight junction structure, which is mediated by tight junction proteins such as occludin, claudin-1, junction adhesion molecule (JAMs), and placental growth factor (PLGF) (48, 49). The presence of PLGF in the serum of patients alters the permeability of the BCSFB by affecting the distribution of tight junction proteins through the VEGFR1-Rho-erk1/2 pathway (50). Studies on the BBB *in vitro* have indicated that inhibition of PLGF in serum can prevent the degradation of tight junctions in endothelial cells, thereby inhibiting the occurrence of brain metastases in GC patients (50, 51). Cancer cells rely on the abundant surrounding blood vessels to proliferate rapidly in the brain, forming the blood tumor barrier (BTB) (52). However, the lack of an *in vitro* BTB model poses a significant challenge to brain tumor research. The recurrence of brain metastasis of GC is a major cause of mortality, likely caused by differences in osmotic concentrations and barriers that hinder drug penetration into the brain, thereby affecting the local availability of tumor drugs in the niche. A number of studies using brain metastasis mouse models have shown that the heterogeneity of the BTB significantly affects the concentration and distribution of drugs in the tumor (53). Currently, clinical treatment methods and strategies are actively being developed to promote the bypass of drugs through the brain barrier and improve drug penetration and increase blood concentration in the brain (54).

In the future, a deeper understanding of the molecular mechanisms and related pathways involved in the process of tumor cells bypassing the BCSFB will pave the way for the development of drugs to treat brain metastasis in GC patients.

### 2.4 Colonization

The colonization of cancer cells and subsequent formation of visible tumors are closely associated with the process of neovascularization, which is critical for the unlimited proliferation of cancer cells. Recent studies have identified several neovascular factors, including vascular endothelial growth factor, transforming growth factor, basic fibroblast growth factor and platelet-derived growth factor, among others (55).

The vascular endothelial growth factor (VEGF) family is a highly specific and critical regulator of colonization. It directly promotes the mitogenic activity of vascular endothelial cells, the growth of vascular endothelium, and vascular permeability. There are six members identified in the VEGF family, including VEGF-A, VEGF-B, VEGF-C, VEGF-D, PLGF and endocrine gland-derived vascular endothelial growth factor (56). Research indicates that VEGF-A is the predominant factor that induces tumor angiogenesis and formation. It is expressed in a wide range of cancers, including GC brain metastasis. The tyrosine kinase receptors (RTKs), VEGFR-2 (KDR), and (fer-like iron deficiency-induced transcription factor) Fit-1 receptors are the three receptors with high affinity to VEGF-A. These receptors are mainly expressed in endothelial cells, with only a small amount expressed in monocytes and hematopoietic cells (57). Bevacizumab, a VEGF inhibitor, has shown significant efficacy in treating GC brain metastasis. Clinical trials have confirmed that adding bevacizumab as a first-line drug with chemotherapy can significantly prolong the median progression-free survival (mPFS) of patients with advanced-GC compared to chemotherapy alone, with tolerable adverse reactions (58).

## 3 Immune checkpoint inhibitors

In recent years, scientists have discovered that the immune system has the potential to eliminate cancers. Currently, ICIs are widely used in experimental research and clinical treatments for various malignancies due to their high specificity, long-lasting immune responses, and long-term survival benefits (59). Among these, ICIs-mediated pathways, such as programmed death protein-1 (PD-1)/programmed death ligand-1 (PD-L1), cytotoxic T-lymphocyte-associated protein 4 (CTLA-4), and lymphocyte activation gene-3 (LAG-3), have been extensively studied for the treatment of GC (**Table 1**).

**Table 1:** Therapies for the treatment of gastric cancers with brain metastasis.

Trial	Phase	Patients (n)	Drugs	ORR,% (95% CI)	PFS, months (95% CI)	mOS,months (95% CI)	Ref.
KEYNOTE-059	II	259	Pembrolizumab	11.6 (8.0-16.1) CPS≥ 1: 15.5 (10.1-22.4)	2.0 (2.0-2.1)	5.6 (4.3-6.9)	(60)
		25	Pembrolizumab plus cisplatin, 5-Fu and capecitabine	60 (38.7-78.9) CPS≥ 1: 68.8 (41.3-89.0)	6.6 (5.9-10.6)	13.8 (8.6-NR) CPS≥ 1: 11.1 (5.4-22.3)	
		31	Pembrolizumab	25.8 (11.9-44.6)	3.3 (2.0-6.0)	20.7 (9.2-20.7)	
KEYNOTE-061	III	196	Pembrolizumab		1.5 (1.4-2.0) CPS≥ 1: 1.5 (1.4-2.0)	9.1 (6.2-10.7) CPS≥ 10: 10.4 (5.9-11.3)	(61)
		199	Paclitaxel		4.1 (3.1-4.2) CPS≥1: 4.1 (3.1-4.2)	8.3 (7.6-9.0) CPS≥ 10: 8.0 (5.1-9.9)	
CheckMate-649	III	789	Nivolumab + chemotherapy	CPS ≥ 5: 60 (55–65)	7.7 (7.1-8.5) CPS ≥ 1: 7.5 (7.0–8.4) CPS ≥ 5: 7.7 (7.0–9.2)	13.8 (12.6-14.6) CPS ≥ 1: 14.0 (12.6–15.0) CPS ≥ 5: 14.4 (13.1–16.2)	(62)
JAVELIN Gastric 100			Avelumab	13.3 (9.3-18.1)	3.2 (2.8-4.1) CPS≥ 1:4.3 (2.9-6.8)	10.4 (9.1-12.0) CPS≥1:14.9 (8.7-17.3)	(63)
			Oxaliplatin + fluoropyrimidine	14.4 (10.3-19.4)	4.4 (4.0-5.5) CPS≥ 1:5.1 (4.2-7.0)	10.9 (9.6-12.4) CPS≥1:11.6 (8.4-12.6)	
ATTRACTION-4	III	362	Nivolumab + chemotherapy	57.5 (52.2-62.6)	10.45 (8.44-14.75)	17.45 (15.67-20.83)	(64)
		362	Chemotherapy	47.8 (42.5-53.1)	8.34 (6.97-9.4)	17.15 (15.18-19.65)	
DESTINY-Gastric01	II	125	T-DXd	51.3 (41.9-60.5)		12.5 (10.3-15.2)	(65)
		62	PC	14.3 (6.6-26.2)		8.9 (6.4-10.4)	
KEYN0TE-811	III	133	KEYTRUDA + Trastuzumab + Fluoropyrimidine and Platinum Chemotherapy	74.4 (66.2–81.6)			(66)
		131	Placebo + Trastuzumab + Fluoropyrimidine and Platinum Chemotherapy	51.9 (43.0–60.7)			
CheckMate-032	I/II	160	Nivolumab 3 mg/kg	12 (5–23)	1.4 (1.2–1.5)	6.2 (3.4–12.4)	(67)
			Nivolumab 1 mg/kg +ipilimumab 3 mg/kg	24 (13–39)	1.4 (1.2–3.8)	6.9 (3.7–11.5)	
			Nivolumab 3 mg/kg + ipilimumab 1 mg/kg	8 (2–19)	1.6 (1.4–2.6)	4.8 (3.0–8.4)	
NCT03409848	II		Nivolumab+trastuzumab +Ipilimumab		3.3 (2.0-6.5)		(68)
			Nivolumab+trastuzumab +FOLFOX		10.7 (6.6-13.1)		
NCT02340975	Ib/II	135	Durvalumab,2L	0 (0-14.2)	1.6 (1.0-1.8)	3.2 (1.7-4.4)	(69)
			Tremelimumab,2L	8.3 (0.2-38.5)	1.7 (0.8-5.3)	7.7 (2.1-13.7)	
			Durvalumab+ tremelimumab, 2L	7.4 (0.9-24.3)	1.8 (1.6-3.3)	1.8 (1.6-3.3)	
			Durvalumab+ tremelimumab, 3L	4.0 (0.1-20.4)	1.8 (1.6-3.5)	1.8 (1.6-1.9)	
NCT01585987	II	18	Ipilimumab	1.8 7.0	2.9 (1.6-5.2) 4.9 (3.5-6.5)	12.7 (10.5-18.9) 12.1 (9.3-NR)	(70)
NCT03472365	II	48	SHR-1210+CAPOX	65	NR	NR	(71)
		19	SHR-1210+ apatinib				
CS1001–101	I	29	CS1001 + XELOX	62			(72)
NCT02954536	II	37	Pembrolizumab, trastuzumab plus	91 (78-97)	13.0 (8.59-NR)	27.17 (18.85-NR)	(73)
SOPHIA	III	266	Margetuximab+ chemotherapy	25	5.7 (5.22-6.97)	21.6 (18.86-24.05)	(74)
		270	Trastuzumab+ pembrolizumab	14	4.4 (4.14-5.45)	19.8 (17.54-22.28)	
CheckMate-577	III	532	Nivolumab				(75)
		262	placebo				
LEAP-005	II	31	Lenvatinib+ pembrolizumab	10 (2-26)	2.5 (1.8-4.2)	5.9	(76)
CP-MGAH22-05	Ib/II	95	Margetuximab+ chemotherapy	18.48 (11.15-27.93)	2.73 (1.61-4.34)	12.48 (9.07-14.09)	(77)
EPOC1706	II	29	Lenvatinib+ pembrolizumab	69 (49-85)	7.1 (5.4-13.7)		(78)
				CPS≥ 1: 84 (60-97)	CPS≥ 1: 9.1		
EGONIVO	Ib	25	Regorafenib+nivolumab	44 (24.4-65.1)	5.6 (2.7-10.4)	12.3 (5.3-NR)	(79)
NCT02013154	Ib/IIa	34	DKN-01+pembrolizumab	DKK1 high: 50 DKK1 low: 0	DKK1 high: 22.1 weeks	DKK1 high: 31.6 weeks	(80)
					DKK1 low: 5.9 weeks	DKK1 low: 17.4 weeks	
UMIN-CTR	I/II	43	Nivolumab combined with paclitaxel plus ramucirumab	37.2 (23.0-53.5)	5.1 (4.5-6.5)	13.1 (8.0-16.6)	(81)
					CPS≥ 1: 6.4 (4.2-7.9)	CPS ≥1: 13.8 (8.0-19.5)	
POLARIS-02	Ib/II	58	Toripalimab	12.1 TMB-H: 33.3	1.9 TMB-H:2.5	4.8 TMB-H: 14.6	(82)
				TMB-H, PD- L1+: 33.3	TMB-H, PD- L1: 2.7	TMB-H, PD- L1+:12.1	
KEYNOTE-062	III	250	Pembrolizumab	37.2	CPS≥ 1: 6.4 (5.7-7.0)	11.1 (9.2-12.8)	(83)
				CPS≥ 1: 37.2	CPS≥ 10: 6.1 (5.3-6.9)	CPS≥ 10: 10.8 (8.5-13.8)	
				CPS≥ 10: 37.8			
		256	Chemotherapy	14.8 CPS≥ 10: 25.0	CPS≥ 1: 2.0 (1.5-2.8)	10.6 (7.7-13.8)	
		257	Pembrolizumab+ chemotherapy	CPS≥ 1: 48.6	CPS≥ 1: 6.9 (5.7-7.3)	12.5 (10.8-13.9) CPS≥ 10: 12.3 (9.5-14.8)	
NCT02937116	Ib	20	Sintilimab+CapeOx		7.5 (6.2-9.4)	NR	(84)
NCT02942329	I	25	Camrelizumab+apatinib	16	2.9 (2.5-4.2)	11.4 (8.6-NR)	(85)
NivoRam	I/II	46	Nivolumab with paclitaxel plus ramucirumab	26.7	2.9	9	(86)

### 3.1 PD-1/PD-L1 inhibitors

PD-1 is expressed as a monomer on the surface of activated T cells, B cells, and monocytes, and functions as an immune molecule that negatively regulates the immune system. During tumor development, the highly expressed PD-L1 molecules on tumor cells bind to the PD-1 and B7.1 receptors on the surface of activated T cells, leading to the differentiation of T cells into depleted T cells or regulatory T cells.

PD-1 antibodies, such as Pembrolizumab, Nivolumab, Toripalimab, Tislelizumab, Camrelizumab, and others, have been studied for treating patients with advanced gastric cancer, including brain metastases. In the 2016 KEYNOTE-012 study, Pembrolizumab showed potential activity, with about 20% of patients experiencing disease remission (87). In the 2017 KEYNOTE-059 study, Pembrolizumab monotherapy demonstrated an OS of 5.6 months, a PFS of 2 months, and an objective remission rate (ORR) of 11.6% (60). In the same year, the ATTRACTION-2 study evaluated Nivolumab, which showed significant survival benefits in patients with advanced gastric cancer and brain metastases, with a mOS of 5.26 months (95%CI, 4.60-6.37) in the monotherapy arm (88). However, the overall effectiveness of PD-1/PD-L1 therapy in GC patients was only 12%, and follow-up showed that the disease may develop brain metastases and progress to highly aggressive forms (64, 89).

### 3.2 CTLA-4 inhibitors

CTLA-4 is a protein receptor found on regulatory T cells that, when bound to the B7 molecule, is capable of forming a negative regulatory immune system for T cells. Additionally, it acts as a switch when it binds to CD86 and CD80 on the surface of antigen-presenting cells (69).

The main anti-CTLA-4 antibodies available are tremelimumab and ipilimumab, which inhibit T-cell activity by targeting CTLA-4. Tremelimumab in combination with durvalumab is commonly used as a second-line treatment for patients with gastric cancer with distant metastases. In a phase Ib/II trial, patients with gastric cancer who received this monoclonal antibody after completing chemotherapy had an mOS of 7.7 months and a PFS of 1.7 months. The ORRs for third-line and second-line combination therapy were 4.0% and 7.4%, respectively. However, the one-year OS rates were only 38.8% and 37%, respectively (90). In CheckMate-032, a phase I/II clinical study, ipilimumab administered as monotherapy (3 mg/kg) in patients with advanced gastric cancer who had progressed on chemotherapy including brain metastases showed an ORR of less than 15% (83). In a subsequent phase II clinical trial (NCT01585987), ipilimumab failed to demonstrate any significant benefits in patients with advanced GC after first-line chemotherapy. Specifically, patients did not experience any survival benefits with this monotherapy as maintenance therapy (91).

### 3.3 LAG-3 inhibitors

Besides PD-1 and CTLA4, various other novel immune checkpoints have been utilized in patients with GC with brain metastases.

In 2022, the FDA approved relatlimab as the third immune checkpoint inhibitor, following PD-1 and CTLA4, for treating melanoma with other metastases (92). Trials NCT03610711, NCT03704077 and NCT03662659 have been conducted to evaluate the efficacy of relatlimab combined with nivolumab for treating GC, with relevant results still pending (93, 94). Researchers have also conducted the SGNTGT-001 study (NCTO4254107) and the HLX301 study (NCT05102214), both targeting TIGIT and PD-L1, to treat patients with advanced GC, including those with brain metastases. These clinical trials are currently awaiting results (95). Additionally, advanced GC patients are expected to benefit from targeting T cell immunoglobulin and mucin-domain containing-3 (TIM-3) and T cell immunoglobulin and ITIM domain (TIGIT) (83).

## 4 Treatment of GC brain metastasis

### 4.1 Surgery

Since 1990, surgical treatment has been recognized as a means to achieve local control of brain tumors and extend survival. Today, surgical treatment remains a critical option for patients with limited brain metastasis.

Operable brain tumors are characterized by the following: gastric cancer patients with brain metastases must have a physical condition that can tolerate surgery; isolated tumors larger than 3cm or tumors requiring pathological diagnosis; tumors with cystic or necrotic edema; and metastatic sites that are easy to remove (96, 97). Surgical resection of brain metastases can significantly reduce the incidence of cancer spread and prolong survival. It is recommended to combine surgery and radiotherapy or chemotherapy for tumor types that are sensitive to these treatments, even in cases where the patient has a single metastatic tumor in the brain (97). Surgery is only recommended for patients with multiple brain metastases if it is necessary to relieve life-threatening compression symptoms.

### 4.2 Whole brain radiotherapy

When patients have severe symptoms such as increased intracranial pressure, meningeal irritation, poor physical activity, and epilepsy and the location of brain metastasis is difficult to determine, WBRT is often the first treatment option for gastric cancer with brain metastasis (98).

In 2020, the National Comprehensive Cancer Network (NCCN) guidelines recommend WBRT after initial chemotherapy for patients with brain metastases without brain symptoms (99). The classic approach to WBRT is to irradiate the whole brain at both field levels in pairs, with radiotherapy doses ranging from 20-40Gy in 5-20 fractions. The standard radiotherapy regimen consists of 30Gy/10 fractions. A higher biological dose of WBRT is 37.5Gy/15 fractions, is available, but this dose can lead to serious complications such as radiation-induced brain damage (100). For patients with a good prognosis for brain metastases (evaluated using the graded prognostic assessment (GPA) index) and for whom stereotactic radiosurgery is not recommended, WBRT is recommended as the primary treatment option, with a total of 30 Gy delivered in 10 fractions. For patients with a poor prognosis for brain metastases, reasonable options include palliative or hospice care, and short-term WBRT (e.g. 20 Gy/5 fractions) for patients with symptomatic brain metastases (100). In contrast to the treatment regimen described above, patients with a single brain metastasis may undergo re-localized field reduction with the additions of 15-20 Gy delivered over 1.5-2 weeks (99).

WBRT has been shown to benefit patients with brain metastases but can also cause complications, with neurocognitive dysfunction being the most common impairment. In a large phase III trial, Brown et al. (101) reported that hippocampal avoidance-WBRT (HA-WBRT) can better protect cognitive function than traditional WBRT. Similarly, avoiding the hippocampus during WBRT has been shown to be closely related to improved neurocognitive function, avoidance of personality changes, and improved quality of life, as mentioned by V. Pareek (102). In addition, the results of phase III trial (NCT02360215) showed that the combined use of memantine after HA-WBRT resulted in a 23.3% deterioration of executive function after 4 months, with declines of 11.5% and 16.4% in learning and memory after 6 months (103). Patients treated with WBRT combined with memantine decreased by 40.4%, 24.7% and 33.3%, respectively. Unfortunately, the RTOG trial showed no significant benefit in terms of mOS and intracranial mPFS from increasing the dose of radiotherapy or changing the segmentation method for patients with brain metastases (104). In addition, researchers have not reached a clinical consensus on WBRT combined with local push radiotherapy, and further clinical exploration is needed.

### 4.3 Stereotactic radiosurgery

The primary treatment of brain metastases was mainly WBRT. However, with the widespread use of CT, SRS has become increasingly popular as it can deliver high-dose radiation to the lesion while sparing the surrounding normal brain tissue. SRS has been shown to have a good therapeutic effect on larger tumors while minimizing radiation complications (105, 106). In recent years, SRS has gradually become the mainstream treatment for metastases due to its advantages over WBRT (107). Researchers have compared the effects and risks of SRS alone versus SRS combined with WBRT in the treatment of multiple brain metastases. The experimental results of NCCTGN0574 indicated that for patients with 2-4 metastatic lesions, SRS alone was associated with less cognitive impairment than SRS combined with WBRT, which significantly reduced immediate recall, delayed recall, and language fluency (108). While the addition of WBRT resulted in little difference in survival rates between patients receiving SRS + WBRT and SRS treatment, it notably improved cognitive function and quality of life in those who received it. In 2014, Yamamoto et al. (109) conducted the JLGK0901 experiment on patients with 2-4 and 5-10 brain metastases, showing no significant difference in adverse reactions and overall survival time between the two groups. Therefore, SRS alone can be considered as a treatment option for multiple brain metastases, regardless of the number of lesions, although its effectiveness may vary depending on the location, size, and number of metastases. According to the guidelines by the American Society for the Treatment of Radiation Oncology (ASTRO) (100), SRS alone is a better choice for patients with fewer than 15 brain metastases from gastric cancer. It should be noted that most studies have been limited to patients with less than 15 brain metastases, and further prospective studies are needed to determine the efficacy of SRS treatment for patients with a larger number of brain metastases.

### 4.4 Chemotherapy

Chemotherapy is the most common treatment for gastric cancer, as it can alleviate systemic symptoms and improve the quality of life. It is a safe and promising treatment for patients with brain metastasis of gastric cancer (38).

At present, the combination of platinum and fluorouracil is the first-line treatment, with oxaliplatin favored over cisplatin due to toxic factors. In a 2022 phase III clinical trials called EXELOX, researchers compared the dual regimen (XELOX: oxaliplatin plus capecitabine) and triple regime (EOX: epirubicin, oxaliplatin plus capecitabine) in treating brain metastasis of gastric cancer. Patients were randomly assigned to receive either regimen at a 1:1 ratio. The PFS of the XELOX regimen group and the EOX regimen group were 5.0 months and 5.5 months, respectively. The median OS time was 12.0 months for both groups. The incidence of grade 3-4 adverse events was 42.2% for XELOX and 72.5% for EOX (110), indicating that the XELOX regimen is superior in PFS and has lower toxicity. Therefore, XELOX regimen is expected to improve the quality of life of patients with brain metastasis of gastric cancer. In addition, the presence of the blood-brain barrier (BBB) plays a pivotal role in maintaining suboptimal concentrations of chemotherapeutic agents within the central nervous system, and more chemotherapeutic drugs are expected to benefit patients in the future.

### 4.5 Immunotherapy

Immune checkpoint inhibitors (ICIs) have made a significant breakthrough in clinical research of advanced gastric cancer and are now considered an important treatment option for patients with brain metastasis of gastric cancer. ICIs can be adminstered as a single drug, or in combination with chemotherapy, radiotherapy, or multiple immune drugs.

#### 4.5.1 Immunotherapy combined with radiotherapy

Combining brain radiotherapy with anti-PD-1 therapy presents a new treatment option for brain metastasis of gastric cancer. In recent years, several patients with advanced solid tumor brain metastases have achieved survival benefits from radiotherapy combined with ICIs (111–113). Compared with radiotherapy alone, patients with brain metastasis of gastric cancer treated with nivolumab and radiotherapy showed a more marked tumor response. The tumor volume of the nivolumab combined with radiotherapy group was reduced by more than 70% compared to the radiotherapy group (114). In a phase I trial, 60 patients with advanced GC who presented with 1-4 brain metastases showed moderate anti-tumor activity after receiving radiotherapy combined with immunotherapy (115). Thus, for patients with brain metastases from gastric cancer, ICIs treatment can increase the tumor sensitivity to radiotherapy (114). However, some researchers believe that after local radiotherapy combined with immunotherapy, brain metastases will regress, i.e., the distant effect (116), which provides great help for subsequent researchers. At present, for patients with brain metastasis of gastric cancer, the optimal timing, treatment sequence and radiotherapy dose of radiotherapy combined with ICIs for patients with brain metastasis of gastric cancer need further investigations in the future (117, 118).

#### 4.5.2 ICIs combined with chemotherapy

Chemotherapy drugs can be toxic to metastatic tumor cells, but their combination with ICIs can have a synergistic effect. CheckMate-649, and ATTRACTION-4 studies have discussed the first-line treatment of gastric cancer brain metastasis with ICIs combined with chemotherapy. These studies have promoted clinical practice in the treatment of gastric cancer brain metastasis.

In the CheckMate-649 study, patients were randomly assigned to the combined group, treated with nivolumab combined with CapeOX, and the control group, treated with FOLFOX chemotherapy alone. The results showed that nivolumab combined with chemotherapy significantly improved mOS (14.4 months vs. 11.1 months, HR=0.71, *P*<0.0001) and mPFS (7.7 months vs. 6.0 months, HR=0.68, *P*<0.0001) in patients with a PD-LI combined positive score (CPS)≥5 (62). Another phase III study, ATTRACTION-4, showed the efficacy and safety of chemotherapy alone as a first-line treatment for brain metastases from gastric cancer by comparing the nivolumab combined with SOX or CapeOX regimen. The mPFS and mOS were 10.45 months vs. 8.34 months and 17.45 months vs. 17.15 months, respectively (64). The above two studies have shown that the immune checkpoint inhibitor nivolumab combined with chemotherapy may become a new standard in the first-line treatment of HER2-negative gastric cancer brain metastasis. In the future, the combination of ICIs and anti-targeted therapy needs to be further explored in the treatment of HER2-positive gastric cancer brain metastases.

#### 4.5.3 ICIs combined with targeted therapy

At present, there are numerous drugs for studying gastric cancer targets. Including Her-2 (also known as ERBB2), vascular Endothelial Growth Factor Receptor2, Claudin18.2, Mesenchymal-epithelial transition factor, and so on (119–121). Approximately 20% of patients with brain metastases from gastric cancer develop amplification or overexpression of the Her-2. Currently, the main drugs used to treat HER2-positive gastric cancer include trastuzumab, lapatinib and neratinib (122).

The combination of trastuzumab and chemotherapy is currently considered standard first-line treatment for patients with this tumor (123). In 2022, the American Society of Clinical Oncology Gastrointestinal Cancer Symposium (ASCO GI) reported the results of the DESTINY-Gastric01 study, which showed that the T-DXd group improved the ORR of patients (51.3% vs 14.3%) and prolonged OS (12.5 and 8.9 months) compared to the PC group (65). With the development of the theory of adding pembrolizumab to HER2-positive patients, several phase I/II studies have been conducted. The EPOC1706 study explored the efficacy and safety of pembrolizumab combined with lenvatinib as first-line or second-line treatment for brain metastasis of GC. The results showed that the ORR of all patients was 69%, the ORR of 14 patients with first-line treatment was 71%, and the median PFS was 7.1 months. Moreover, the trial showed no occurence of grade 4-5 irAEs, and the safety of the scheme is controllable (78). Recent studies have shown that the ORR of PD-1 monoclonal antibody combined with standard first-line regimen can reach 56%-91%, the median PFS is 8.6-13.0 months, and the OS is 19.3-27.2 months (124, 125). The results of KEYN0TE-811 further confirmed the advantages of the combined treatment model. The ORR of patients with first-line pembrolizumab+trastuzumab chemotherapy (cisplatin+fluorouracil or oxaliplatin+capecitabine) was 74.4%, compared to 22.5% in the traditional trastuzumab+ chemotherapy group (*P*<0.001) (66). Among them, 11.3% of patients in the combined treatment group achieved complete remission, while only 3.1% in the control group. The DOR of the two groups was 10.6 months and 9.5 months, respectively. Based on the results of this study, in 2021, the US Food and Drug Administration (FDA) approved pembrolizumab for the first-line treatment of patients with HER-2-positive G/GEJ adenocarcinoma brain metastasis.However, its OS and PFS results have not been published, and the final survival benefit remains to be clarified.

#### 4.5.4 Double ICIs combination

Combining anti-CTLA-4 and anti-PD-1/PD-L1 therapies has also been explored in gastric cancer patients with brain metastasis. In a multicenter phase I/II clinical trial called CheckMate-032, the efficacy and safety of nivolumab alone or in combination with ipilimumab were evaluated in the treatment of advanced gastric cancer (67). A total of 160 patients were randomly treated with nivolumab 3mg/kg q2w, nivolumab 1mg/kg combined with ipilimumab 3mg/kg q21d, or nivolumab 3mg/kg combined with ipilimumab 1mg/kg q21d. The results showed that the ORR of the three groups were 12%, 24% and 8%, respectively, as the primary endpoint, and the 1-year OS rates were 39%, 35% and 24%, respectively. A phase I study of advanced gastric cancer (NCT03409848) showed promising efficacy when trastuzumab was combined with ICIs, leading to improved patient survival time (68). However, the mPFS of ipilimumab was lower compared to the FOLFOXZ group (3.3 months vs. 10.7 months), and the exact efficacy needs further confirmation. In addition, clinical studies of other dual ICIs such as HERIZON-GEA-01, are currently ongoing.

## 5 Tumor vaccine therapy

Tumor vaccines are an emerging direction in immunotherapy, providing exogenous tumor antigens and activating the body’s immune response (126). Major types of tumor vaccines include peptide vaccines, DC vaccines, viral vaccines, and mRNA vaccines (127).

Several tumor vaccines against gastric cancer have been developed, including HER-2-targeted peptide vaccine IMU-131 (HER-Vaxx) (NCT02795988), OTSGC-A24 peptide vaccine (NCT03784040) and MG7-DC vaccine (NCT04567069) (128–130). While there is currently no FDA-approved tumor vaccine for brain metastasis of gastric cancer, this field shows a broad prospect and is a current research hotspot. Oncolytic virus vaccines represent a new approach to immunotherapy that has emerged in recent years. Research has shown that oncolytic viruses can be engineered to express PD-L1 inhibitors, which can continuously activate the anti-tumor effect of T cells (131). In addition, mRNA vaccines are another type of tumor vaccine. In theory, mRNA vaccines can specifically target brain metastatic tumor cells, reflecting the concept of precision medicine (132). The efficacy of a novel mRNA vaccine (NEO-PV-01) has been evaluated in melanoma and bladder cancer and has shown promising results, with efficacy comparable to or even exceeding that of existing ICI monotherapy (NCT02897765) (133). Currently, clinical trials are investigating the efficacy of mRNA vaccines in treating brain metastasis of gastric cancer (NCT03468244), and the results are highly anticipated (134). In summary, cellular immunotherapy and tumor vaccines are essential strategies for achieving individualized precision immunotherapy of tumors in addition to ICIs.

## 6 Immune-related adverse events

Immunotherapy has shown promise in providing long-lasting remission in certain cancer patients. However, it can also leads to specific toxicities known as irAEs. These events can occur in any system and are caused by the upregulation of the inflammatory response resulting from the release of immune cells by ICIs.

PD-1/PD-L1 inhibitors are associated with common irAEs, including interstitial pneumonia, hyperthyroidism, hypothyroidism, hypopituitarism, hepatitis, pancreatitis, myositis, colitis, nephritis, and severe skin reactions (135, 136). irAEs are also observed in patients receiving advanced gastric cancer treatment with ICIs, as seen in other cancers. irAEs were slightly higher when chemotherapy was combined with ICIs compared to other regimens. For instance, in the DESTINY-Gastric01 study, 85.6% of patients in the T-DXd group had grade 3 and above adverse events, while 56.5% of patients in the PC group had controllable safety (65). In the KEYNOTE-062 study, for example, the incidence of grade 3-5 irAEs was 14% and 35%. In the study, the incidence of irAEs at grade 3 or higher was 6% in both the combination therapy and pembrolizumab groups, with 24% of patients in the former group and 21% in the latter experiencing irAEs (83). To mitigate toxicity from irAEs, cytokines such as IL-6 and GM-CSF have been proposed as potential targets for decoupling the immune response (137, 138). It is worth noting that some studies suggest an uncertain correlation between irAEs and the clinical effectiveness of immunotherapy, which requires further investigation (139).

## 7 Conclusion

Brain metastases are a significant cause of mortality and morbidity in patients with gastric cancer and other malignancies, substantially affecting their survival and quality of life. However, the molecular mechanisms and functions underlying brain metastases are not well understood. Brain metastases do not arise solely from a population of cancer cells; rather, they result from a complex interplay of cellular interactions and molecular signaling pathways. The underlying processes are multi-faceted and intricate, and unique features of the brain, including the blood-brain barrier, the intracerebral microenvironment, and the blood supply, may respond differently to associated pathways and neuroendocrine changes. Currently, *in vitro* cellular assays and mouse models of brain metastasis provide a theoretical foundation, but clinical studies in humans are necessary to further explore these findings. There is ongoing development of screening tools and techniques that can facilitate the timely and precise detection of brain metastases. With the emergence of immune checkpoint inhibitors and other novel therapies, they are expected to play a crucial role in the combined treatment of brain metastases, enhancing quality of life and prolonging survival for high-risk patients.

## Author contributions

YZ: Conceptualization, Data curation, Writing – original draft. MZ: Project administration, Resources, Writing – review & editing. CL: Investigation, Software, Visualization, Writing – original draft. WK: Conceptualization, Data curation, Writing – original draft. YH: Data curation, Project administration, Writing – original draft.
